# Early natural killer cell counts in blood predict mortality in severe sepsis

**DOI:** 10.1186/cc10501

**Published:** 2011-10-21

**Authors:** David Andaluz-Ojeda, Verónica Iglesias, Felipe Bobillo, Raquel Almansa, Lucía Rico, Francisco Gandía, Ana Ma Loma, Concepción Nieto, Rosa Diego, Epifanio Ramos, Mercedes Nocito, Salvador Resino, Jose M Eiros, Eduardo Tamayo, Raul Ortiz de Lejarazu, Jesús F Bermejo-Martin

**Affiliations:** 1 Critical Care Medicine Service, Hospital Clínico Universitario-SACYL/SEMICYUC, Avda Ramón y Cajal 3, E-47005, Valladolid, Spain; 2 Immunology Section and Infection & Immunity Medical Investigation Unit (IMI), Microbiology and Immunology Service, Hospital Clínico Universitario-IECSCYL, Avda Ramón y Cajal 3, E-47005 Valladolid, Spain; 3 Anesthesiology Service. Hospital Clínico Universitario, Avda Ramón y Cajal 3, E-47005 Valladolid, Spain; 4 Laboratory of Molecular Epidemiology of Infectious Diseases, National Centre of Microbiology, Instituto de Salud Carlos III, Ctra Majadahonda-Pozuelo Km, E-2200 Majadahonda, Madrid, Spain

## Abstract

**Introduction:**

Host immunity should play a principal role in determining both the outcome and recovery of patients with sepsis that originated from a microbial infection. Quantification of the levels of key elements of the immune response could have a prognostic value in this disease.

**Methods:**

In an attempt to evaluate the quantitative changes in the status of immunocompetence in severe sepsis over time and its potential influence on clinical outcome, we monitored the evolution of immunoglobulins (Igs) (IgG, IgA and IgM), complement factors (C3 and C4) and lymphocyte subsets (CD4+ T cells, CD8+ T cells, B cells (CD19+) and natural killer (NK) cells (CD3-CD16+CD56+)) in the blood of 50 patients with severe sepsis or septic shock at day 1, day 3 and day 10 following admission to the ICU.

**Results:**

Twenty-one patients died, ten of whom died within the 72 hours following admission to the ICU. The most frequent cause of death (*n *= 12) was multiorgan dysfunction syndrome. At day 1, survivors showed significantly higher levels of IgG and C4 than those who ultimately died. On the contrary, NK cell levels were significantly higher in the patients who died. Survivors exhibited a progressive increase from day 1 to day 10 on most of the immunological parameters evaluated (IgG, IgA, IgM, C3, CD4+, CD8+ T cells and NK cells). Multivariate Cox regression analysis, including age, sex, APACHE II score, severe sepsis or septic shock status and each one of the immunological parameters showed that NK cell counts at day 1 were independently associated with increased risk of death at 28 days (hazard ratio = 3.34, 95% CI = 1.29 to 8.64; *P *= 0.013). Analysis of survival curves provided evidence that levels of NK cells at day 1 (> 83 cells/mm^3^) were associated with early mortality.

**Conclusions:**

Our results demonstrate the prognostic role of NK cells in severe sepsis and provide evidence for a direct association of early counts of these cells in blood with mortality.

## Introduction

Severe sepsis (acute organ dysfunction secondary to infection) and septic shock (severe sepsis plus hypotension not reversed by fluid resuscitation) are major healthcare problems that affect millions of individuals around the world each year, killing one in four (and often more) and increasing in incidence [1-3]. Similarly to polytrauma, acute myocardial infarction and stroke, the early initiation of therapy once severe sepsis is established is likely to influence the patient's prognosis. In consequence, early identification of individuals at risk for bad outcomes is dramatically important in this condition [4].

Because sepsis originates from a microbial infection, host immunity should play a principal role in determining both outcome and recovery. A protective role of naturally produced immunoglobulin G (IgG) in sepsis was described previously [5]. The participation of cellular immunity in this disease is poorly understood, and the available data are controversial [6-8].

Identifying quantitative alterations in key humoral and cellular parameters could have a prognostic value in this condition. In an attempt to evaluate the quantitative changes in the status of immunocompetence in severe sepsis over time and its potential influence on clinical outcome, we monitored levels of blood of Immunoglobulins (IgG, IgA and IgM), complement factors (C3 and C4), lymphocyte subpopulations (T, B and natural killer (NK) cells) in 50 consecutive patients with a diagnosis of severe sepsis or septic shock at three moments during their hospitalization in the ICU. Our measurement of these parameters provide a first assessment of the status of both humoral and cellular arms of the immune response, and their evaluation is easily available in the vast majority of hospitals with critical care medicine units.

## Materials and methods

### Patients

#### Inclusion criteria

Patients ages 18 years and older with a diagnosis of severe sepsis or septic shock upon admission to our ICU were prospectively and consecutively recruited from January 2010 to January 2011. The first day following ICU admission was considered day 1 in the analysis. 'Sepsis' was defined as suspected infection in the presence of two or more systemic inflammatory response syndrome criteria [9]. 'Severe sepsis' was defined as sepsis plus sepsis-induced organ dysfunction or tissue hypoperfusion [10]. Sepsis-induced hypotension was defined as systolic blood pressure (SBP) < 90 mmHg, mean arterial pressure < 70 mmHg or SBP decrease > 40 mmHg or < 2 SD below normal for age in the absence of other causes of hypotension. 'Septic shock' was defined as hypotension (SBP < 90 mmHg) despite adequate fluid resuscitation (> 1,500 ml) or the use of vasoactive agents [10]. Severity of illness was assessed on the basis of two scores: the Acute Physiology and Chronic Health Evaluation II (APACHE II) score for the first 24 hours following diagnosis [11] and the Sequential Organ Failure Assessment (SOFA) score [12].

#### Exclusion criteria

Exclusion criteria were the presence of immunodeficiency or concomitant immunosuppressive therapy, pregnancy, do not resuscitate status and cardiac arrest. Approval of the study protocol for both the scientific and ethical aspects was obtained from the Scientific Committee for Clinical Research of our hospital. Informed consent was obtained directly from each patient or his or her legal representative before enrolment.

### Microbiological diagnostics

Standard cultures in biological samples guided by the presumptive source of the septic insult were performed to assess the presence of bacterial and fungal infection [13], along with detection of a urinary antigen test for *Legionella pneumophila or Streptococcus pneumoniae*. In addition, two consecutive identifications by the LightCycler SeptiFast Test MGRADE (Roche Molecular Diagnostics, Pleasanton, CA, USA) real-time PCR in blood were also considered a positive result. Potentially contaminant microorganisms were not considered.

### Immunological laboratory workup

A serum sample was collected from each patient at day 1, day 3 and day 10 following admission to the ICU. IgG, IgM, IgA, C3 and C4 levels in serum were measured by using a Dade Behring BN II System nephelometer (Siemens Healthcare Diagnostics, Deerfield, IL, USA). A blood sample was collected in parallel by using tubes containing ethylenediaminetetraacetic acid. Quantification of lymphocytes subpopulations was performed by using BD Trucount tubes (BD Biosciences, San Jose, CA, USA) for enumeration of mature human T (CD3+), B (CD19+), helper/inducer T (CD3+CD4+), suppressor/cytotoxic T (CD3+CD8+) and NK (CD3-CD16+CD56+) lymphocytes by using a BD FACSCalibur 4-color flow cytometer (342975; BD Biosciences).

### Statistical analysis

Comparison of immune parameters levels based upon mortality were performed using the Mann-Whitney *U *test. Differences in the levels of immune parameters over the observation period were assessed using the Wilcoxon signed-rank test. We determined the HR and 95% CI by Cox regression analysis, which was used to assess the impact of independent variables on mortality over time. Multivariate analysis, including age, sex, APACHE II score, severe sepsis or septic shock status and each one of the immunological parameters, was performed. These variables were checked for colinearity prior to inclusion in the regression models using Tolerance and Variance Inflation Factor. We determined the occurrence of death by using Kaplan-Meier curves. Groups were compared by using the log-rank test (Mantel-Haenzel). Logarithmic concentrations of the immune parameters evaluated were employed in the regression analysis to satisfy the linearity assumption. All statistical tests were two-sided, and *P *< 0.05 was considered significant. Data analysis was performed using SPSS for Windows version 15.0 software (SPSS, Chicago, IL, USA).

## Results

### Clinical characteristics of the patients

The majority of the patients of our cohort were elderly and male, with nonsurvivors being older than survivors (Table 1). The most common comorbidities were cardiovascular disease, diabetes and arterial hypertension. Chronic obstructive pulmonary disease was present in many patients who ultimately died during their ICU hospitalization. Eight patients had an antecedent of solid organ neoplasia with criteria of cure. None of these patients were received chemotherapy or showed evidence of metastasis at the time of admission. Septic shock was the most frequent cause of admission to the ICU, mostly in the group who eventually died. The principal suspected source of infection was the lower respiratory tract. The presence of a microorganism was documented in 72% of the cases, with a balanced proportion of Gram-positive and Gram-negative bacteria. Two patterns of mortality were observed in our cohort: a group of patients (*n *= 10) died within the 72 hours following admission to the ICU and a group of patients (*n *= 11) who died later than day 3. All but one of the patients who died presented at admission with status of septic shock. The most frequent cause of death (*n *= 12) was multiorgan dysfunction syndrome. Refractory shock, refractory hypoxemia and cardiovascular events were the causes of the death in the remaining fatal cases. Five nonsurvivors and three survivors received corticosteroids as part of their severe sepsis management. All these patients presented with septic shock, and steroids were administered after the first 24 hours of their ICU stay (hydrocortisone (50 mg/6 hours or 100 mg/8 hours intravenously).

**Table 1:** Demographics and clinical characteristics of the patients based upon ICU mortality

Demographic and clinical characteristics	Survivors (*n *= 29)	Nonsurvivors (*n *= 21)	Total population (*n *= 50)	Survivors vs nonsurvivors *P *value
Age (years)	66.0 (20.0)	72.0 (14)	68.5 (19.2)	0.048
Males	18 (62%)	14 (66.6%)	32 (64%)	n.s.
APACHE II score at ICU admission	18.0 (15.0)	23.0 (10.0)	20.5 (13.2)	n.s.
SOFA score at ICU admission	7.0 (6.0)	9.0 (2.0)	8.0 (4.0)	n.s.
Comorbidities				
Cardiovascular disease	8 (26.6%)	7 (35%)	15 (30%)	n.s.
COPD	2 (6.6%)	6 (30%)	8 (16%)	0.025
Chronic renal failure or dialysis	2 (6.6%)	2 (10%)	4 (8%)	n.s.
Diabetes mellitus types 1 and 2	10 (33.3%)	7 (35%)	17 (34%)	n.s.
Alcohol abuse	2 (6.6%)	1 (5%)	3 (6%)	n.s.
Hypertension	12 (41.3%)	7 (33.3%)	19 (38%)	n.s.
Neoplasia	3 (10%)	5 (25%)	8 (16%)	n.s.
Obesity	2 (6.6%)	1 (5%)	3 (6%)	n.s.
Diagnostic at ICU admission				
Severe sepsis	11 (36.6%)	1 (5%)	12 (24%)	0.007
Septic shock	18 (62%)	20 (95.2%)	38 (76%)	0.007
Presumed source of infection				
Lower respiratory tract/pneumonia	14 (46.6%)	9 (45%)	23 (46%)	n.s.
Urogenital	6 (20%)	3 (15%)	9 (18%)	n.s.
Intra-abdominal	2 (6.6%)	3 (15%)	5 (10%)	n.s.
Catheter- or device-related	1 (3.3%)	1 (5%)	2 (4%)	n.s.
Skin (soft tissues)	2 (6.6%)	1 (5%)	3 (6%)	n.s.
Prosthesis	2 (6.6%)	0 (0%)	2 (4%)	n.s.
Central nervous system	1 (3.3%)	0 (0%)	1 (2%)	n.s.
Other/unknown	2 (6.8%)	4 (19%)	6 (12%)	n.s.
Documented microbial agent				
Gram-positive	8 (26%)	6 (30%)	14 (28%)	n.s.
Gram-negative	10 (34.4%)	9 (42.8%)	19 (38%)	n.s.
Fungi	2 (6.6%)	1(5%)	3 (6%)	n.s.

Continuous variables are expressed as medians (IQR). All other data are raw numbers (%). Proportions were compared using the Χ^2 ^test or Fisher's exact test where appropriate. APACHE II: Acute Physiology and Chronic Health Evaluation II; COPD: chronic obstructive pulmonary disease; SOFA: sequential organ failure assessment score; n.s. = not significant.

### Comparison of immunological parameters levels based upon outcome

At day 1, survivors showed significantly higher levels of IgG and C4 than those who died during hospitalization in the ICU (Table 2). On the contrary, NK cell absolute counts were significantly higher in the group of patients who died. The relative concentrations of NK cells in blood (percentage of total lymphocytes) were also higher in the group of nonsurvivors (median (IQR): survivors = was 9 (12.5) and nonsurvivors = 20 (24.5); *P *< 0.05). Comparison of immune parameters levels at day 3 evidenced higher levels of IgG in those patients who survived (Table 2). No differences were found at this moment in the course of the disease for the other parameters compared (Table 2). When comparisons were repeated considering only those patients with septic shock (*n *= 38), the same results were obtained (Additional file 1). Survivors exhibited a progressive increase from day 1 to day 10 on most of the immunological parameters evaluated (IgG, IgA, IgM, C3, CD4+, CD8+ T cells and NK cells) (Additional file 2).

**Table 2:** Comparison of immunological parameters based upon ICU mortality

	ICU mortality from day 1 (*N *= 50)			ICU mortality from day 3 (*N *= 40)			ICU mortality from day 10 (*N *= 37)		
Immunological parameters	Survivors (*n *= 29)	Nonsurvivors (*n *= 21)	*P *value	Survivors (*n *= 29)	Nonsurvivors (*n *= 11)	*P *value	Survivors (*n *= 28)	Nonsurvivors (*n *= 9)	*P* value
IgG (mg/dl)	844.0 (447.5)	616.0 (442.5)	0.035	932.0 (371.0)	685.0 (538.0)	0.007	1,080.0 (602.2)	764.0 (1068.0)	n.s.
IgA (mg/dl)	240.0 (128.0)	184.0 (167.0)	n.s.	280.0 (144.0)	240.0 (209.0)	n.s.	334.0 (164.0)	257.0 (580.8)	n.s.
IgM (mg/dl)	53.0 (47.0)	60.0 (53.0)	n.s.	70.0 (85.0)	67.0 (61.0)	n.s.	79.5 (66.5)	42.0 (86.0)	n.s.
C3 (mg/dl)	101.5 (52.3)	79.0 (60.5)	n.s.	108.0 (55.0)	110.0 (89.0)	n.s.	135.0 (49.0)	106.0 (81.0)	n.s.
C4 (mg/dl)	24.0 (19.0)	17.0 (16.0)	0.029	24.0 (16.5)	28.0 (22.0)	n.s.	32.0 (18.0)	20.0 (10.0)	n.s.
CD3+ T (cells/mm^3^)	417.0 (706.5)	419.0 (585.0)	n.s.	639.5 (636.3)	472.0 (415.0)	n.s.	774.0 (463.5)	561.0 (552.0)	n.s.
CD4+ T (cells/mm^3^)	288.0 (474.5)	198.0 (400.0)	n.s.	372.5 (525.8)	366.0 (423.0)	n.s.	519.0 (339.0)	347.0 (355.0)	n.s.
CD8+ T (cells/mm^3^)	114.0 (258.0)	156.0 (231.0)	n.s.	249.5 (199.0)	120.0 (145.0)	n.s.	287.0 (296.0)	204.0 (314.0)	n.s.
CD4+ CD8+ T (cells/mm^3^)	5.0 (12.0)	6.0 (13.5)	n.s.	5.0 (9.0)	6.5 (24.0)	n.s.	12.0 (18.3)	6.0 (54.0)	n.s.
LB (cells/mm^3^)	103.0 (165.5)	184.0 (281.0)	n.s.	163.0 (125.0)	127.0 (168.0)	n.s.	118.0 (151.0)	92.0 (131.0)	n.s.
NK (cells/mm^3^)	56.8 (59.5)	98.0 (392.0)	0.050	59.5 (72.5)	42.0 (73.0)	n.s.	86.0 (78.0)	92.0 (38.0)	n.s.

Data are expressed as medians (IQR). n.s. = not significant. Normal values in adult healthy individuals are as follows: immunoglobulin G (IgG) = 870 to 2,180 mg/dl, IgA = 117 to 420 mg/dl; IgM = 60 to 220 mg/dl; CD3+ T cells = 690 to 2,540 cells/mm^3^; CD4+ T cells = 410 to 1,590 cells/mm^3^; CD8+ T cells = 190 to 1,140 cells/mm^3^; CD4+ CD8+ T cells = not available; B lymphocytes (LB) = 90 to 660 cells/mm^3^; natural killer (NK) cells = 90 to 590 cells/mm^3^; complement factor 3 (C3) = 50 to 120 mg/dl; complement factor 4 (C4) = 14 to 70 mg/dl.

### Immune parameters and prediction of mortality

**Figure 1:**
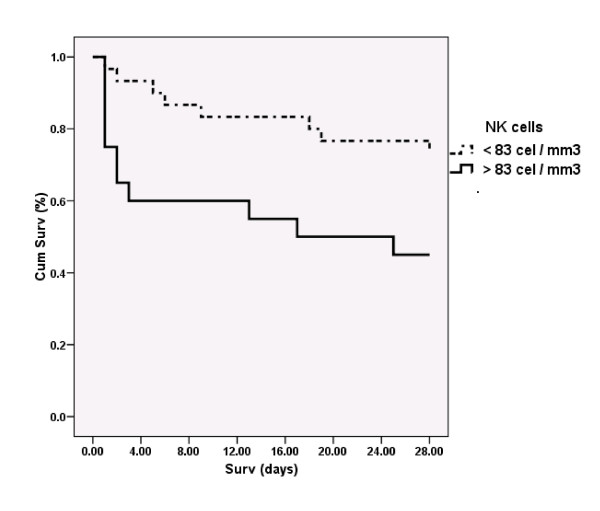
**Kaplan-Meier curves**. Deciles from percentile 10 to percentile 90 of natural killer (NK) cell counts measured at day 1 were calculated and used to compare survival times in those patients with low or high concentrations of NK cells in their blood. The first decile shows significant differences between groups based upon the log-rank test (Mantel-Haenzel) and was used as the cutoff (percentile 60 (83 cells/mm^3^)). Outcome for this analysis was time until death. Time was censored at day 28. Cum Surv: cumulative survivors; NK: natural killer.

Multivariate Cox regression analysis showed that NK cell counts at day 1 were independently associated with increased risk of death at 28 days. The median outcome of death at day 28 was 3.34 (95% CI = 1.29 to 8.64; *P *= 0.013). Analysis of the survival curves evidenced that levels of NK cells at day 1 (> 83 cells/mm^3^) were associated with early mortality (Figure 1).

When multivariate regression analysis was repeated considering only septic shock patients, NK cell counts at day 1 remained a risk factor for mortality (HR = 3.20, 95% CI = 1.23 to 8.35; *P *= 0.017). IgG levels at day 1 showed a protective association with increased survival at day 28 which was close to statistical significance (HR = 0.10, (95% CI = 0.01 to 1.15; *P *= 0.065).

## Discussion

Our results provide evidence that differences in the systemic levels of a number of key host immunity elements in patients with severe sepsis influence their final outcome. Compared to those patients who survived, septic patients who died showed lower levels of IgG and C4, along with higher levels of NK cells, in the first 24 hours following admission to the ICU. Comparisons between fatal cases and survivors, as well as the results of our regression analysis, suggest that NK cell counts at day 1 are associated with increased risk of mortality in patients who present to the ICU with severe sepsis. The role of these cells in sepsis is controversial [14]. NK-cell depletion increases survival and decreases systemic levels of cytokines in experimental models of sepsis [7,15-21]. In humans, available data derived from patients in the ICU are scarce, and some of them diverge with the results derived from animal models. Gogos *et al*. [22] found increased absolute counts of NK cells in sepsis caused by community-acquired pneumonia. Giamarellos-Bourboulis *et al*. [8] reported improved survival in patients with severe Gram-negative sepsis and high NK counts. NK cells have sophisticated biological functions [23], participating with antigen presentation cells and T cells in the cellular response against pathogens. NK cells are key actors in innate immunity and as a consequence should play an important role in the very early moments of sepsis. In addition, NK cells could release high amounts of proinflammatory cytokines such as IFN-γ or immunosuppressive agents, such as IL-10, thus promoting tissue damage and interfering with the development of the adaptive immune response against the causative microbial agent [23].

When we performed a subanalysis considering only those patients with septic shock, NK cell counts at day 1 were still associated with increased risk of mortality. On the contrary, IgG levels at day 1 showed a protective trend close to statistical significance in the regression analysis. Taccone *et al*. [5] reported low concentrations of IgG as a common finding in patients with community-acquired septic shock. In their study, patients with hypo-IgG had greater vasopressor requirements, were more likely to develop acute lung injury and/or acute respiratory distress syndrome and had higher mortality. Studies with larger numbers of patients are necessary to confirm the protective trend of IgG in this disease.

The relatively lower levels of C4 observed in patients who died in our study could be a consequence of consumption of this complement in severe sepsis. In agreement with our results, Nakae *et al*. [24] found significantly lower levels of C3 and C4 in septic patients who did not survive than in those who did. Analysis of the variations over time in the levels of the immunological parameters evaluated showed that most survivors exhibited a progressive increase from day 1 to day 10 in most of the parameters. This result provides evidence for a compromised host response during the very first moments of severe sepsis (at least from the quantitative point of view) which improves at the latter stages of the disease in these patients. Interestingly, nonsurvivors failed to show significant increments in the levels of the immune parameters measured over time. These findings warrant further work with larger cohorts of patients to evaluate the inclusion of immunological parameters as part of the severity scores in sepsis. A limitation of our study is the definition employed for NK cells (CD3-CD16+CD56+ lymphocytes), since it did not include a subset of CD16- NK cells, which account for a minor percentage of total NK cells (5% to 10%). Nonetheless, the definition used in this work identified the largest population of these cells.

## Conclusions

Our results demonstrate the prognostic role of NK cells (defined as CD3-CD16+CD56+ lymphocytes) in severe sepsis, proving a direct association of early blood counts of these cells with mortality. Further studies including functional analysis of NK cells are needed to confirm their exact role in the pathogenesis of this disease.

## Key messages

◆ Being that sepsis originates from a microbial infection, host immunity should play a principal role in determining both outcome and recovery. Identifying quantitative alterations in key humoral and cellular parameters could have a prognostic value in this disease.

◆ Compared to survivors, septic patients who did not survive showed lower levels of IgG and C4 and higher levels of NK cells in blood in the first 24 hours following admission to the ICU.

◆ Kaplan Meier curves and Cox regression analysis demonstrate the prognostic role of NK cells (defined as (CD3-CD16+CD56+) lymphocytes) in severe sepsis, evidencing a direct association of early blood counts of these cells with mortality.

## Abbreviations

APACHE II: Acute Physiology and Chronic Health Evaluation II; C3: complement factor 3; C4: complement factor 4; CD: cluster differentiation; COPD: chronic obstructive pulmonary disease; HR: hazard ratio; IFN-γ: interferon γ; Ig: immunoglobulin; IL: interleukin; LB: B lymphocyte: NK: natural killer; PCR: polymerase chain reaction; SBP: systolic blood pressure; SOFA: Sequential Organ Failure Assessment.

## Competing interests

The authors declare that they have no competing interests.

## Authors' contributions

DAO, FB, FG, ROL and JFBM assisted in the design of the study, coordinated patient recruitment, analysed and interpreted the data and assisted in writing the paper. SR, ET, JME, RA and MN assisted in the analysis and interpretation of data and in writing the report. VI, LR, AML, CN, RD and ER performed the laboratory work. All authors read and approved the final manuscript for publication.

## Supplementary Material

Additional file 1 **Comparison of immunological parameters based upon ICU mortality in patients with septic shock**. Data are medians [IQR]. n.s = not significant. Normal values in healthy adults are as follows: immunoglobulin G (IgG) = 870 to 2,180 mg/dl; IgA = 117 to 420 mg/dl; IgM = 60 to 220 mg/dl; CD3+ T cells = 690 to 2,540 cells/mm^3^; CD4+ T cells = 410 to 1,590 cells/mm^3^; CD8+ T cells = 190 to 1,140 cells/mm^3^; CD4+CD8+ T cells = not available; B lymphocytes (LB) = 90 to 660 cells/mm^3^; natural killer (NK) cells = 90 to 590 cells/mm^3^; complement factor 3 (C3) = 50 to 120 mg/dl; complement factor 4 (C4) = 14 to 70 mg/dl. Click here for file

Additional file 2 **Comparison of immunological parameter levels over time**. Changes in the levels of immune parameters over time in survivors and nonsurvivors were assessed using the Wilcoxon signed-rank test. The results are expressed as medians (IQR) of the increments (day 3 - day 1) and (day 10 - day 1). IgG = immunoglobulin G; IgM = immunoglobulin M; IgA = immunoglobulin A; C3: complement factor 3; C4: complement factor 4; NK cells: natural killer cells; LB = B lymphocytes. Click here for file
