# Supplementary material for: Early natural killer cell counts in blood predict mortality in severe sepsis
Source: Crit Care. 2011 Oct 21;15(5):R243. doi: 10.1186/cc10501 (PMC3334794; doi:10.1186/cc10501)
Supplement: Additional file 1 — Comparison of immunological parameters based upon ICU mortality in patients with septic shock. Data are medians [IQR]. n.s = not significant. Normal values in healthy adults are as follows: immunoglobulin G (IgG) = 870 to 2,180 mg/dl; IgA = 117 to 420 mg/dl; IgM = 60 to 220 mg/dl; CD3+ T cells = 690 to 2,540 cells/mm3; CD4+ T cells = 410 to 1,590 cells/mm3; CD8+ T cells = 190 to 1,140 cells/mm3; CD4+CD8+ T cells = not available; B lymphocytes (LB) = 90 to 660 cells/mm3; natural killer (NK) cells = 90 to 590 cells/mm3; complement factor 3 (C3) = 50 to 120 mg/dl; complement factor 4 (C4) = 14 to 70 mg/dl. [file cc10501-S1.DOC]

|  | **ICU mortality from day 1** | | ***p*** |
| --- | --- | --- | --- |
|  | **Survivors (n=18)** | **Non survivors**  **(n=20)** |  |
| **A) Immunological parameters** (d1) **n = 38** | | | |
| **IgG (mg/dl)** | **840.5 [338.3]** | **594.5 [408.8]** | ***0.047*** |
| **IgA (mg/dl)** | 260.0 [158.3] | 178.5 [170.0] | n.s |
| **IgM (mg/dl)** | 49.5 [84.8] | 51.0 [50.5] | n.s |
| **C3 (mg/dl)** | 104.0 [54.8] | 77.5 [65.3] | n.s |
| **C4 (mg/dl)** | **21.0 [15.5]** | **16.5 [15.0]** | ***0.023*** |
| **CD3 (+) T (cel/mm^3^)** | 416.0 [801.3] | 405.5 [523.8] | n.s |
| **CD4 (+) T (cel/mm^3^)** | 332.0 [505.0] | 263.5 [407.0] | n.s |
| **CD8 (+) T (cel/mm^3^)** | 102.5 [262.3] | 147.0 [238.0] | n.s |
| **CD4 (+) CD8 (+) T (cel/mm^3^)** | 6.5 [13.5] | 6.0 [13.8] | n.s |
| **LB (cel/mm^3^)** | 85.0 [82.5] | 191.0 [279.3] | n.s |
| **NK (cel/mm^3^)** | **55.5 [66.5]** | **97.0 [431.0]** | ***0.035*** |
|  | **ICU mortality from day 3** | | ***p*** |
|  | **Survivors (n=18)** | **Non survivors**  **(n=10)** |  |
| **A) Immunological parameters** (d3) **n = 28** | | | |
| **IgG (mg/dl)** | **939.0 [291.5]** | **597.0 [467.8]** | ***0.005*** |
| **IgA (mg/dl)** | 300.0 [156.0] | 205.5 [219.5] | n.s |
| **IgM (mg/dl)** | 69.5 [104.3] | 61.5 [57.0] | n.s |
| **C3 (mg/dl)** | 104.5 [51.5] | 102.5 [83.8] | n.s |
| **C4 (mg/dl)** | 25.5 [15.3] | 28.0 [21.0] | n.s |
| **CD3 (+) T (cel/mm^3^)** | 639.5 [941.0] | 477.0 [485.8] | n.s |
| **CD4 (+) T (cel/mm^3^)** | 479.0 [551.0] | 367.0 [435.5] | n.s |
| **CD8 (+) T (cel/mm^3^)** | 256.5 [252.0] | 119.5 [183.0] | n.s |
| **CD4 (+) CD8 (+) T (cel/mm^3^)** | 7.0 [15.5] | 4.0 [33.0] | n.s |
| **LB (cel/mm^3^)** | 188.0 [170.5] | 146.5 [179.5] | n.s |
| **NK (cel/mm^3^)** | 63.5 [55.5] | 40.0 [80.3] | n.s |
|  | **ICU mortality from day 10** | | ***p*** |
|  | **Survivors (n=17)** | **Non survivors**  **(n=8)** |  |
| **A) Immunological parameters** (d10) **n = 25** | | | |
| **IgG (mg/dl)** | 1070.0 [637.5] | 548.0 [1107.8] | n.s |
| **IgA (mg/dl)** | 353.0 [136.0] | 247.0 [620.5] | n.s |
| **IgM (mg/dl)** | 105.0 [96.0] | 39.5 [81.5] | n.s |
| **C3 (mg/dl)** | 127.0 [46.5] | 96.5 [53.8] | n.s |
| **C4 (mg/dl)** | 29.0 [18.0] | 19.5 [7.5] | n.s |
| **CD3 (+) T (cel/mm^3^)** | 774.0 [457.3] | 692.0 [655.0] | n.s |
| **CD4 (+) T (cel/mm^3^)** | 507.5 [336.3] | 422.5 [384.8] | n.s |
| **CD8 (+) T (cel/mm^3^)** | 207.0 [163.3] | 256.0 [324.8] | n.s |
| **CD4 (+) CD8 (+) T (cel/mm^3^)** | 12.0 [23.3] | 7.5 [60.0] | n.s |
| **LB (cel/mm^3^)** | 118.5 [130.8] | 93.5 [102.8] | n.s |
| **NK (cel/mm^3^)** | 75.5 [128.0] | 94.0 [47.8] | n.s |
