# Supplementary material for: Early natural killer cell counts in blood predict mortality in severe sepsis
Source: Crit Care. 2011 Oct 21;15(5):R243. doi: 10.1186/cc10501 (PMC3334794; doi:10.1186/cc10501)
Supplement: Additional file 2 — Comparison of immunological parameter levels over time. Changes in the levels of immune parameters over time in survivors and nonsurvivors were assessed using the Wilcoxon signed-rank test. The results are expressed as medians (IQR) of the increments (day 3 - day 1) and (day 10 - day 1). IgG = immunoglobulin G; IgM = immunoglobulin M; IgA = immunoglobulin A; C3: complement factor 3; C4: complement factor 4; NK cells: natural killer cells; LB = B lymphocytes. [file cc10501-S2.DOC]

|  | **Paired comparisons by Wilcoxon test**  ***P ( d1-d3)***  ***P ( d1-d10)*** | | | |
| --- | --- | --- | --- | --- |
|  | **Δ (d3 –d1)** | ***p***  ***(d1 vs d3)*** | **Δ (d10 –d1)** | ***p***  ***(d1 vs d10)*** |
| **A) Immunological parameters**  **in survivors** | **Survivors from day 3**  **n = 29** |  | **Survivors from day 10**  **n = 28** |  |
| **IgG (mg/dl)** | 20 (217) | n.s | **270** (**305**) | ***0.001*** |
| **IgA (mg/dl)** | **29** (**54**) | ***0.007*** | **116** (**120**) | ***0.035*** |
| **IgM (mg/dl)** | **20** (**66**) | ***0.001*** | **31.5** (**86**) | ***0.008*** |
| **C3 (mg/dl)** | 4 (33.0) | n.s | **43** (**52**) | ***0.001*** |
| **C4 (mg/dl)** | 0 (17.5) | n.s | 1.5 (19) | n.s |
| **CD3 (+) T (cel/mm^3^)** | **177** (**475**) | ***0.024*** | **383** (**406**) | ***0.010*** |
| **CD4 (+) T (cel/mm^3^)** | **98** (**281**) | ***0.013*** | **230** (**383**) | ***0.031*** |
| **CD8 (+) T (cel/mm^3^)** | **52** (**219**) | ***0.050*** | **160** (**131**) | ***0.004*** |
| **CD4 (+) CD8 (+) T (cel/mm^3^)** | 0 (6.0) | n.s | **7** (**15**) | ***0.007*** |
| **LB (cel/mm^3^)** | 32 (154) | n.s | 39 (71) | n.s |
| **NK (cel/mm^3^)** | 2 (60) | n.s | **39.5** (**80**) | ***0.035*** |
| **A) Immunological parameters in fatal cases** | **Fatal cases from day 3**  **n = 11** |  | **Fatal cases from day 10**  **n = 9** |  |
| **IgG (mg/dl)** | -58 (230) | n.s | -17 (516) | n.s |
| **IgA (mg/dl)** | -3 (41) | n.s | -29 (136) | n.s |
| **IgM (mg/dl)** | -12 (15) | n.s | -11 (60) | n.s |
| **C3 (mg/dl)** | 6 (74) | n.s | 91 (43) | n.s |
| **C4 (mg/dl)** | 2 (9) | n.s | 1 (8) | n.s |
| **CD3 (+) T (cel/mm^3^)** | 63 (641) | n.s | -139 (1339) | n.s |
| **CD4 (+) T (cel/mm^3^)** | 76 (158) | n.s | -12 (696) | n.s |
| **CD8 (+) T (cel/mm^3^)** | 36 (232) | n.s | 2 (398) | n.s |
| **CD4 (+) CD8 (+) T (cel/mm^3^)** | 1.5 (17) | n.s | -4 (13) | n.s |
| **LB (cel/mm^3^)** | -35 (137) | n.s | -83 (190) | n.s |
| **NK (cel/mm^3^)** | -9 (46) | n.s | 35 (149) | n.s |
